# Phenotypic Assessment of Clinical Escherichia coli Isolates as an Indicator for Uropathogenic Potential

**DOI:** 10.1128/msystems.00827-22

**Published:** 2022-11-29

**Authors:** A. E. Shea, A. E. Frick-Cheng, S. N. Smith, H. L. T. Mobley

**Affiliations:** a Department of Microbiology and Immunology, University of Michigan Medical School, Ann Arbor, Michigan, USA; University of California Davis

**Keywords:** UPEC, UTI, clinical isolates, pathogenesis, virulence

## Abstract

For women in the United States, urinary tract infections (UTIs) are the most frequent diagnosis in emergency departments, comprising 21.3% of total visits. Uropathogenic Escherichia coli (UPEC) causes ~80% of uncomplicated UTIs. To combat this public health issue, it is vital to characterize UPEC strains as well as to differentiate them from commensal strains to reduce the overuse of antibiotics. It has been challenging to determine a consistent genetic signature that clearly distinguishes UPEC from other E. coli strains. Therefore, we examined whether phenotypic data could be predictive of uropathogenic potential. We screened 13 clinical strains of UPEC, isolated from cases of uncomplicated UTI in young otherwise healthy women, in a series of microbiological phenotypic assays using UPEC prototype strain CFT073 and nonpathogenic E. coli strain MG1655 K-12 as controls. Phenotypes included adherence, iron acquisition, biofilm formation, human serum resistance, motility, and stress resistance. By use of a well-established experimental mouse model of UTI, these data were able to predict the severity of the bacterial burden in both the urine and bladders. Multiple linear regression using three different phenotypic assays, i.e., growth in minimal medium, siderophore production, and type 1 fimbrial expression, was predictive of bladder colonization (adjusted *R*^2^ = 0.6411). Growth in *ex vivo* human urine, hemagglutination of red blood cells, and motility modeled urine colonization (adjusted *R*^2^ = 0.4821). These results showcase the utility of phenotypic characterization to predict the severity of infection that these strains may cause. We predict that these methods will also be applicable to other complex, genetically redundant, pathogens.

**IMPORTANCE** Urinary tract infections are the second leading infectious disease worldwide, occurring in over half of the female population during their lifetime. Most infections are caused by uropathogenic Escherichia coli (UPEC) strains. These strains can establish a reservoir in the gut, in which they do not cause disease but, upon introduction to the urinary tract, can infect the host and elicit pathogenesis. Clinically, it would be beneficial to screen patient E. coli strains to understand their pathogenic potential, which may lead to the administration of prophylactic antibiotic treatment for those with increased risk. Others have proposed the use of PCR-based genetic screening methods to detect UPEC strains and differentiate them from other E. coli pathotypes; however, this method has not yielded a consistent uropathogenic genetic signature. Here, we used phenotypic characteristics such as growth rate, siderophore production, and expression of fimbriae to better predict uropathogenic potential.

## INTRODUCTION

Half of women will experience at least one urinary tract infection (UTI) in their lifetime (1). These ubiquitous infections cause five billion dollars in associated health care costs in the United States and annually affect 150 million women worldwide (2, 3). While a variety of bacteria cause UTIs, the predominant etiological pathogen is uropathogenic Escherichia coli (UPEC), which causes over 80% of uncomplicated cases (4, 5). Therefore, to combat this public health issue, it is vital to fully understand and characterize individual UPEC strains, and clinically, it is beneficial to differentiate UPEC from commensal E. coli.

UPEC is part of the extraintestinal pathogenic E. coli (ExPEC) grouping, which encompasses any E. coli that causes disease outside of the gut (6, 7). However, UPEC resides in both the gut, likely as a reservoir, and the urinary tract, which serves as the site of active infection (8, 9). Unlike other E. coli pathotypes, which can be identified by specific virulence gene repertoires, UPEC has incredible genetic heterogeneity (10, 11) and encodes a wide variety of virulence factors: up to six virulence-associated iron acquisition systems, six toxins, and 13 different adhesins (3, 12–14). Some strains produce almost all these virulence factors, while others produce only a select few. One of the few conserved virulence factors is the highly studied type 1 fimbria; however, commensal isolates such as K-12 also encode this adhesin (15, 16). Furthermore, E. coli strains that cause asymptomatic bacteriuria (ABU) are genetically similar to strains that cause symptomatic infection (17, 18).

A few studies have attempted to develop a diagnostic PCR assay to differentiate between ExPEC and diarrheagenic strains (19, 20). One study showed if a strain carried *fyuA*, *chuA*, *vat*, and *yfcV*, it was 10 times more likely to be a UPEC or neonatal meningitis E. coli (NMEC) isolate (19). However, the authors of that study were unable to differentiate between UPEC and NMEC. Moreover, only 58% of their UPEC cohort carried *vat* and 69% carried *yfcV*, leaving a relatively large population of UPEC strains overlooked.

Another study sought to differentiate between noninvasive UPEC (causing ABU or cystitis) and invasive UPEC (causing pyelonephritis or bacteremia) and found that *papG2* was highly enriched in the invasive strains (21). Although this was an important finding, there is a need to differentiate between ABU- and cystitis-causing strains, especially given that current clinical guidelines state that ABU should not be treated with antibiotics (22–24). Indeed, ABU strains have been proposed as a treatment to prevent symptomatic cystitis (25, 26). Therefore, there is clearly a need to distinguish ABU strains from those that elicit pathogenesis. Interestingly, a different study found that while carriage of virulence factors did not correlate with robust murine bladder colonization, the gene expression profile of these strains did correlate (10). Furthermore, it was also found that higher levels of Lipocalin-2 in the urine of patients also correlated (10). Together, these results imply that bacterial behavior might be more indicative of virulence potential.

UPEC encounters different ecological niches, including the gut and the periurethral area of the host (27, 28). This wide tissue tropism obfuscates which virulence factors are required to infect the urinary tract as opposed to factors used for these other niches, and UPEC strains carry functionally redundant genes. For example, iron acquisition is essential for host colonization and UPEC encodes up to four different siderophores and two different heme receptors (29–31), and deleting a single system is not sufficient to ablate fitness (31). UPEC strains typically encode any number of combinations of these systems; a particular virulence factor is not vital, but the combination is important to achieve uropathogenesis. Consequently, this makes UPEC identification via a single gene, or even several genes, difficult.

To address this, we hypothesized that measuring phenotypic experimental outcomes, rather than genotyping, will better predict infectivity. We characterized 13 clinical UPEC isolates (32, 33) and compared their behavior to that of the well-characterized UPEC type strain CFT073 and intestinal commensal isolate K-12. We performed 18 *in vitro* phenotypic assays associated with known virulence mechanisms of UPEC and correlated the results with an experimental mouse model of UTI. We were able to model the bacterial burden in both the urine and bladders via multiple linear regression using three different phenotypic assays each. Growth in minimal medium, siderophore production, and type 1 fimbrial expression were predictive of bladder colonization (adjusted *R*^2^ = 0.6411), while growth in *ex vivo* human urine, hemagglutination of red blood cells, and motility modeled urine colonization (adjusted *R*^2^ = 0.4821). These results indicated the utility of phenotypic characterization to reduce the complexity of diverse, redundant genomes of UPEC isolates to predict the severity of infection.

## RESULTS

### UPEC strains display diverse sugar metabolism efficiencies and iron acquisition abilities.

**FIG 1:**
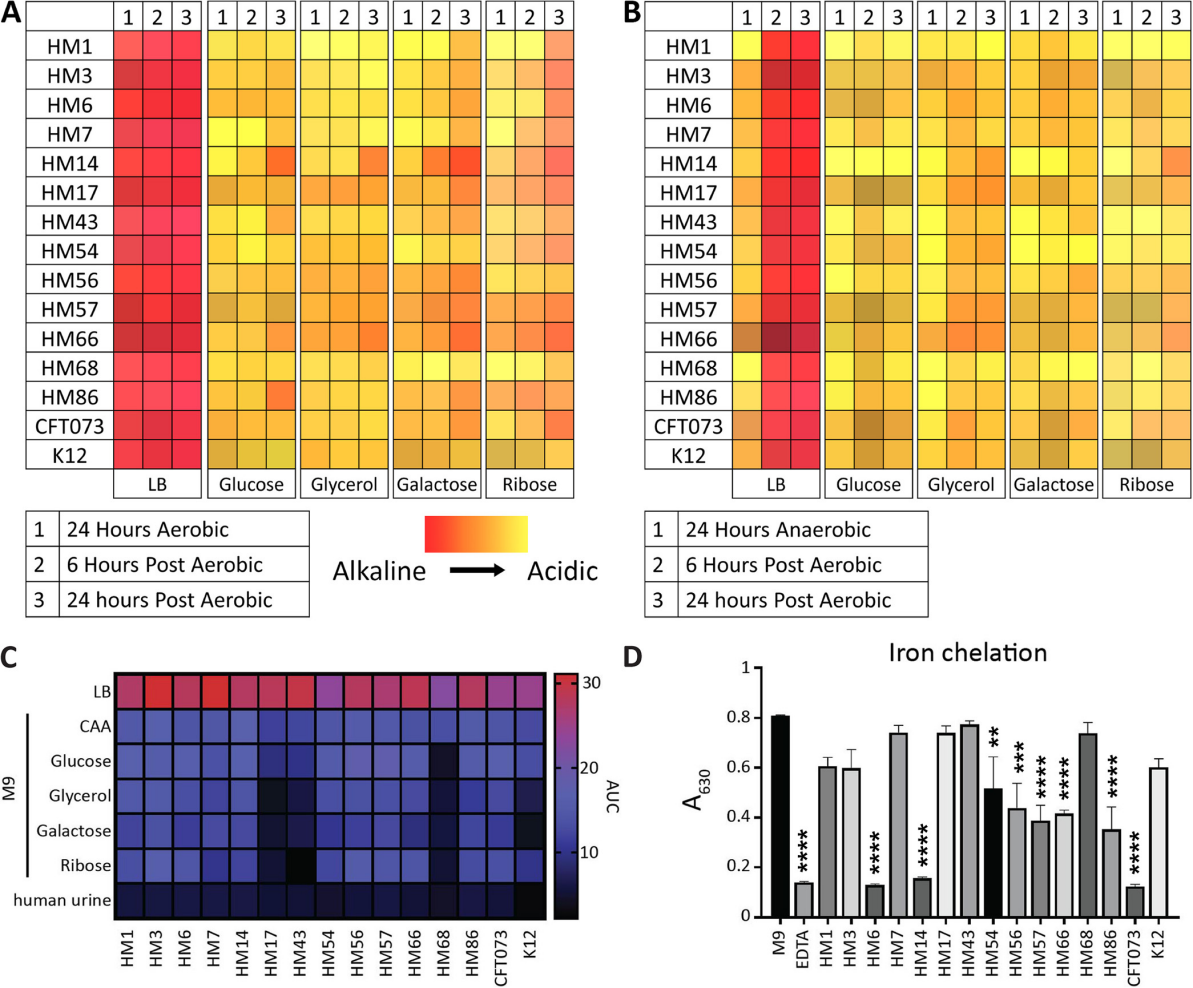
Three UPEC isolates display growth defects on sugar carbon sources and minimal chelation ability. UPEC isolates were spotted from overnight cultures onto LB agar plates containing the different indicated carbon sources and 0.1% phenol red pH indicator. (A and B) Plates were initially incubated at 37°C under either aerobic (A) or anaerobic (B) conditions. Plates were then transitioned to the benchtop for additional time point observation. The colors represent the hues displayed on the agar, with more red indicating more alkaline and yellow indicating acidic end products. (C) UPEC strains were inoculated 1:100 from overnight cultures into different medium types. Strains were incubated at 37°C with aeration for 24 h, and the OD_600_ was recorded every 15 min. The AUC was calculated and is visually represented. Data displayed are a mean result of six biological replicates. (D) Strains were cultured overnight, with shaking at 37°C, in M9 minimal medium supplemented with 0.4% glucose. One hundred microliters of supernatants was combined with 100 μL of chrome azurol S (CAS) shuttle solution and incubated at room temperature for 30 min. Ten millimolar EDTA served as a positive control, and M9 medium only served as a negative control. After 30 min, the absorbance at 630 nm was read. Data are displayed as the mean result of four biological replicates, with error bars indicating the standard error of the mean (SEM). One-way analysis of variance (ANOVA) was performed using Dunnett’s multiple-test correction in comparison to the M9 control. **, *P < *0.01; ***, *P < *0.005; ****, *P < *0.0001.

Rapid growth and metabolic flexibility are key for UPEC survival and infection (33–35). Therefore, we monitored the metabolic switch from anaerobic to aerobic conditions by using phenol red as a pH indicator to model the switch from the gut to the bladder environment. Both anaerobic respiration and fermentation result in lactic acid secretion, thus lowering the local pH and turning the bacterial colony yellow. Aerobic respiration leads to the production of carbon dioxide and water, yielding a red/pink color. Under both aerobic and anaerobic conditions, the clinical isolates were tested on each of four carbohydrates (glucose, glycerol, galactose, or ribose) as a preferred primary carbon source in Luria broth (LB) to mimic anaerobic fermentative conditions (Fig. 1A and B). We observed that on LB plates under aerobic conditions, all strains performed aerobic respiration (Fig. 1A). Under anaerobic conditions, all strains performed fermentation but quickly switched to aerobic respiration after 6 h in an aerobic environment (Fig. 1B). However, the introduction of carbohydrates delayed the switch to aerobic respiration (Fig. 1B) and likely encouraged utilization of glycolysis and the pentose phosphate pathway. Only when the carbohydrate was depleted did bacteria switch over to exclusively amino acid-based metabolic processes, as evidenced by a deepening shift from orange to red only after 24 h of postaerobic incubation.

Our previous study (36) showed that global gene expression in LB recapitulates the transcriptome during human UTI. Pooled human urine can also recapitulate a similar expression profile for specific subsets of genes, such as those that control iron acquisition. Our lab also found that UPEC prefers amino acids as a carbon source in both human and murine UTI (33, 36, 37). We wanted to investigate whether there were any strain-specific differences in growth when carbohydrates were used as a sole carbon source, as opposed to amino acids, and if there were any differences in carbohydrate preference among clinical UPEC isolates. Thus, we analyzed growth in LB, pooled human urine, and M9 minimal medium with five different sole carbon sources, including Casamino Acids (CAA), glucose, glycerol, galactose, or ribose (see Fig. S1A to G in the supplemental material). These individual data were used to calculate the area under the curve (AUC) to better display differences between strains (Fig. 1C). Unsurprisingly, we found robust growth in LB for all E. coli clinical isolates as well as CFT073 and K-12 (Fig. 1C). However, we observed a remarkable defect in K-12 compared to all UPEC isolates when cultured in human urine (Fig. S1G); potentially, growth in urine could differentiate between E. coli strains that can infect the urinary tract. Strains HM17, HM43, and HM68 had restricted growth on all sugar carbon sources (Fig. 1C). However, these strains had robust growth when given CAA as a sole carbon source, as did the rest of the UPEC strains (Fig. 1C). This result further corroborates the studies that show that UPEC prefers amino acids as the optimal carbon source *in vivo* (33, 37).

10.1128/msystems.00827-22.1 FIG S1 Growth of recent UPEC isolates CFT073 and K-12 in LB (A), M9 minimal medium with casamino acids (CAA) (B), glucose (C), glycerol (D), galactose (E), or ribose (F) as a sole carbon source, and pooled *ex vivo* human urine (G). Growth curves show averages of results of six biological replicates, and error bars indicate the SEM. (H) The indicated strains were cultured overnight in LB, and 5 μL of culture was spotted on CAS agar. The color change from blue to orange indicates iron chelation presumably due to siderophore production. An orange halo around the colony is due to diffusion of secreted siderophores. Download FIG S1, TIF file, 1.9 MB. Copyright © 2022 Shea et al. 2022 Shea et al. https://creativecommons.org/licenses/by/4.0/ This content is distributed under the terms of the Creative Commons Attribution 4.0 International license.

Iron, a vital nutrient required for bacterial growth, is highly restricted within the host (38, 39). Most UPEC strains encode several different iron acquisition systems that include the synthesis of siderophores to acquire iron sequestered by the host (31, 40). We assessed siderophore production of these clinical isolates using the chrome azurol S (CAS) assay (41). Strains were cultured overnight in iron-poor M9 medium, and then their supernatants were incubated with CAS dye, which is conjugated with Fe^3+^. When Fe^3+^ is chelated from the dye, it changes from blue to red, resulting in a loss of absorbance at 630 nm. We observed variable levels of CAS activity between strains. Strains CFT073, HM6, and HM14 had strong CAS activity, with chelation of the dye at levels similar to that of the positive control EDTA (Fig. 1D). In fact, these three strains, in addition to HM86, had nonsignificant differences from the positive control EDTA. Six of the strains, HM54, HM56, HM57, HM66, HM68, and HM86, displayed more modest CAS activity (Fig. 1D), though still significantly different from that of the medium-alone control. The remaining five UPEC isolates, strains HM3, HM7, HM17, HM43, and HM68, as well as K-12 had no significant CAS activity (Fig. 1D). Apart from E. coli K-12, it was surprising to observe this apparent lack of activity from strains that are pathogenic and that all encode siderophore systems. Therefore, we adjusted the assay and cultured the strains directly on agar plates containing CAS. While the CAS agar does not result in quantitative data, it is more sensitive to siderophore production since signal builds as the colonies grow and secrete siderophore (41). Using CAS agar, we detected siderophore production in all strains, including K-12 (Fig. S1H).

### Surface structure composition varies between isolates, resulting in unique motility, biofilm, and morphology phenotypes.

**FIG 2:**
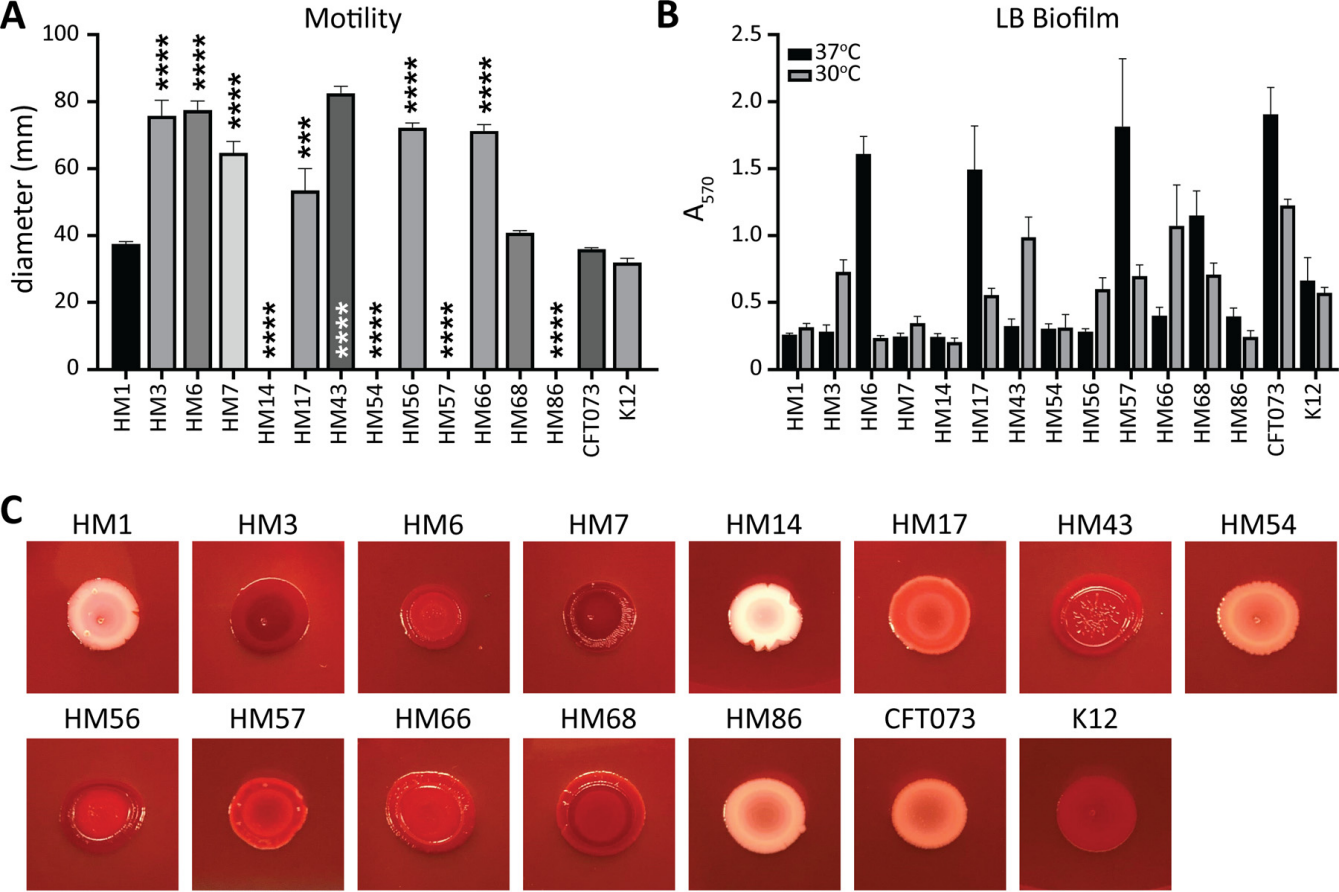
Some UPEC isolates are unable to swim or form biofilms *in vitro*. (A) Motility was assessed by stabbing clinical isolates into semisolid agar and incubating them at 30°C for 16 h. Measured diameters are shown in millimeters with error bars indicating the SEM. Bars represent the average result of biological replicates (*n* = 3). One-way ANOVA was performed with Dunnett’s multiple-test correction in comparison to CFT073. ***, *P < *0.005; ****, *P < *0.0001. (B) The ability of clinical UPEC isolates to form biofilms in LB was determined at both 37°C (black) and 30°C (gray). Bar height represents the average absorbance (570 nm) after staining with crystal violet of four biological replicates, and error bars indicate the SEM. (C) Overnight liquid cultures were spotted onto LB agar plates containing Congo red dye and incubated for 48 h at 30°C.

Uropathogens use motility to ascend the ureters from the bladder to the kidneys and for directed movement toward nutrient-rich areas (42–44). To assess the motility potential of our clinical collection of UPEC strains, we conducted a motility assay in semisoft agar at 30°C (Fig. S2A). After 16 h of incubation, the swimming diameter was measured (Fig. 2A). Interestingly, four strains were nonmotile under these conditions: HM14, HM54, HM57, and HM86 (Fig. 2A). On the other hand, HM3, HM6, HM7, HM43, HM56, and HM66 were hypermotile in comparison to both CFT073 and K-12 (Fig. 2A).

10.1128/msystems.00827-22.2 FIG S2 (A) Representative images of swimming motility assays of the indicated strains. (B) Biofilm formation of clinical UPEC isolates CFT073 and K-12 in pooled *ex vivo* human urine at either 30°C or 37°C. Biofilms were stained with crystal violet and quantified through absorbance at 570 nm. Bars are an average of results of four biological replicates, and error bars indicate the SEM. Download FIG S2, TIF file, 1.1 MB. Copyright © 2022 Shea et al. 2022 Shea et al. https://creativecommons.org/licenses/by/4.0/ This content is distributed under the terms of the Creative Commons Attribution 4.0 International license.

While the ability to move between organ sites is important, so is the ability to establish a permanent community. Uropathogens do this through cell-to-cell interactions, both on abiotic structures such as catheters and within eukaryotic cells as intracellular bacterial communities (IBCs) (45–47). We tested biofilm formation in the clinical isolates at both 30°C and 37°C using a microtiter plate assay and crystal violet, which will stain both matrix and bacteria nonspecifically (Fig. 2B). In LB at 37°C, only HM6, HM17, HM57, and HM68 produced biofilm similar to that of CFT073 (Fig. 2B). It is notable that at 37°C, we observed the biggest differences between CFT073 and K-12. At 30°C, HM3, HM43, HM66, and CFT073 produced the largest biofilm communities (Fig. 2B). Urine biofilms were barely above background detectable levels (Fig. S2B). In addition to examining biofilm potential via crystal violet staining, we also assessed curli formation through growth on a medium containing Congo red dye (Fig. 2C). This dye binds curli, which aid in biofilm formation: curli-positive bacteria appear red, and curli-negative colonies appear white (48). HM14 displayed a unique morphology that very clearly excluded the Congo red dye and exhibited a mucoid phenotype (Fig. 2C). HM3, HM7, HM56, HM68, and K-12 all displayed a very dark red color (Fig. 2C). Many of the strains displayed moderately red or pink colors, indicating a spectrum of curli presence among these uropathogenic strains under these conditions.

### Type 1 fimbrial expression varies greatly among clinical isolates.

**FIG 3:**
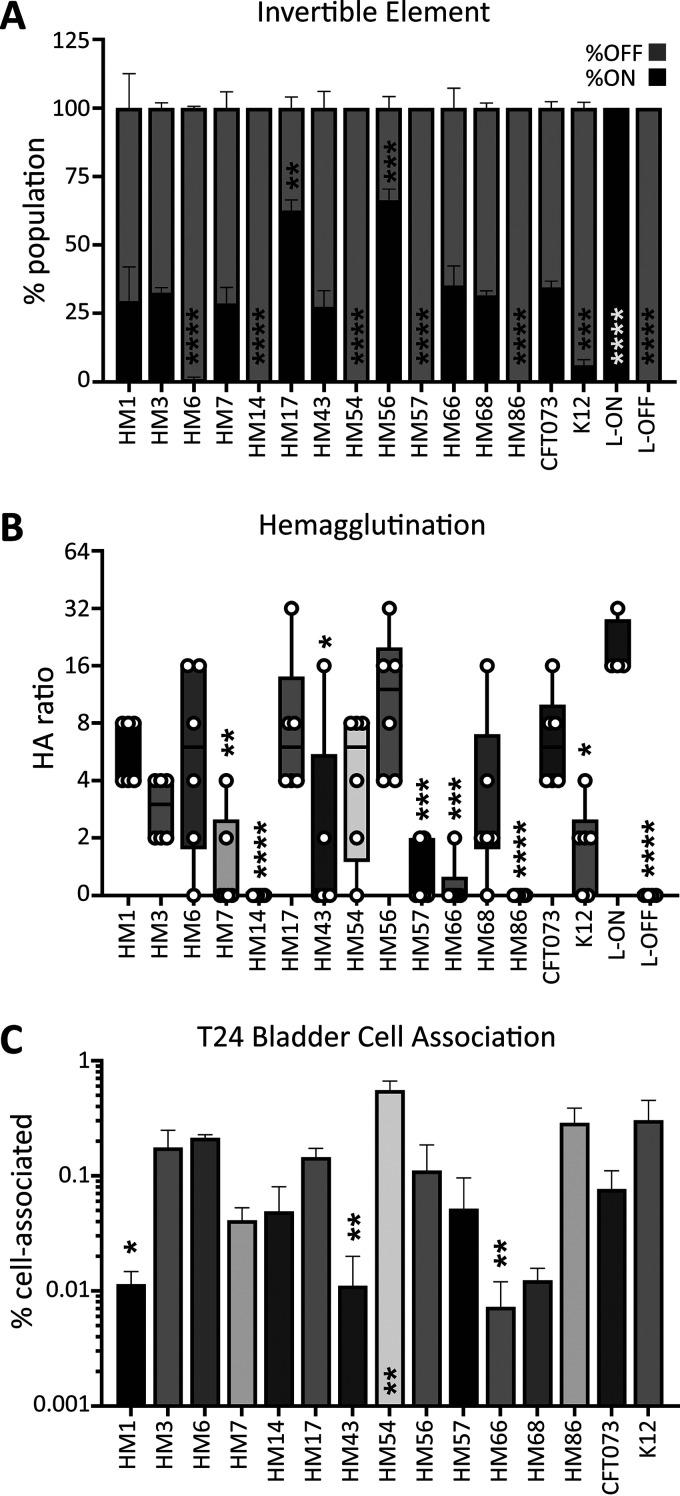
HM17 and HM56 demonstrate increased type 1 fimbrial expression concordant with hemagglutination (HA) and adherence phenotypes. (A) The invertible element (IE) PCR assay was used to quantify the orientation of the *fimS* IE as either on or off. The stacked bar height indicates the mean of the population with *fim* on (black) and *fim* off (gray). Data represent the results of three biological replicates with the SEM shown. CFT073 L-on and L-off constructs were used as controls in which 100% of the total population should be either *fim* on or *fim* off. (B) HA assays were performed with guinea pig erythrocytes by utilizing bacterial titers in a 2-fold serial dilution series, indicated by the HA ratio. Box and whisker plots denote the quartiles and ranges of the data from six biological replicates, shown by the individual points. CFT073 L-on and L-off strains were used as controls. L-on can hemagglutinate at high ratios (1:32), indicating that fewer bacteria are required to produce the phenotype. (C) Clinical UPEC strains were assessed for their ability to adhere to the T24 bladder cell line. Cell-associated bacteria were enumerated after 1 h of coculture with cell monolayers; the bars represent the mean result of four biological replicates with error bars indicating the SEM. The data shown represent the normalized output-to-input ratios as a percentage. One-way ANOVA was performed with Dunnett’s multiple-test correction in comparison to CFT073. *, *P < *0.05; **, *P < *0.01; ***, *P < *0.005; ****, *P < *0.0001.

Adherence is a critical factor during UTI. Specifically, the use of type 1 fimbriae to bind the uroepithelium is essential to withstand the flow of urine (49). These fimbriae are phase variable via an invertible element (50). We utilized a PCR-based assay to determine whether UPEC strains had their invertible elements in the on or off orientation (Fig. S3A) (51). We assessed the percentage of the total bacterial population with the invertible element in the on position after being cultured statically at 37°C, a condition that has previously been shown to induce switching to the on orientation (51). HM17 and HM56 had over 50% of the invertible element in the on position, while HM6, HM14, HM54, HM57, and HM86 had nearly undetectable *fim*-on populations (Fig. 3A).

An alternate method to determine the expression and functionality of bacterial fimbriae is by use of a hemagglutination assay (16). This quantifies UPEC adherence to guinea pig erythrocytes. Bacteria were serially diluted before the addition of erythrocytes; a higher ratio indicates stronger type 1 fimbrial expression. HM1, HM6, HM17, HM56, and CFT073 all exhibited strong hemagglutination phenotypes (Fig. 3B). HM14 and HM86 had no ability to agglutinate red blood cells under these assay conditions (Fig. 3B). This phenotype is type 1 fimbria specific, and the addition of mannose was sufficient to inhibit all hemagglutination (Fig. S3B).

10.1128/msystems.00827-22.3 FIG S3 (A) Representative image of an agarose gel displaying the result of an invertible element PCR assay. Bands representing *fimS* in the off and on positions are labeled. (B) Representative image of a hemagglutination assay. The bottom row shows that the addition of 1% mannose reverses the phenotype seen above, indicating that type 1 fimbriae are the adhesin responsible for the phenotype. Download FIG S3, TIF file, 1.5 MB. Copyright © 2022 Shea et al. 2022 Shea et al. https://creativecommons.org/licenses/by/4.0/ This content is distributed under the terms of the Creative Commons Attribution 4.0 International license.

In addition to the type 1 fimbriae, UPEC employs an arsenal of other adhesins to bind epithelial cells (52). To quantify the contribution of all these systems, we added bacteria to T24 bladder cells at a multiplicity of infection (MOI) of 100 and incubated them for 1 h before determining the number of cell-associated CFU. The ability of clinical isolates to associate with cells ranged widely, from 0.09% (HM66) to 0.75% (HM54), in comparison to input CFU (Fig. 3C). Interestingly, nonpathogenic K-12 had higher cell association than CFT073 (Fig. 3C). Also of note, while HM14 does not appear to have type 1 fimbriae (Fig. 3A and B), it was able to successfully cell associate *in vitro* (Fig. 3C). This demonstrates that there are other, likely surface-expressed, structures that aid in UPEC-cell association (52).

### A decrease in host cell metabolic activity is directly linked to beta-hemolysis of red blood cells.

**FIG 4:**
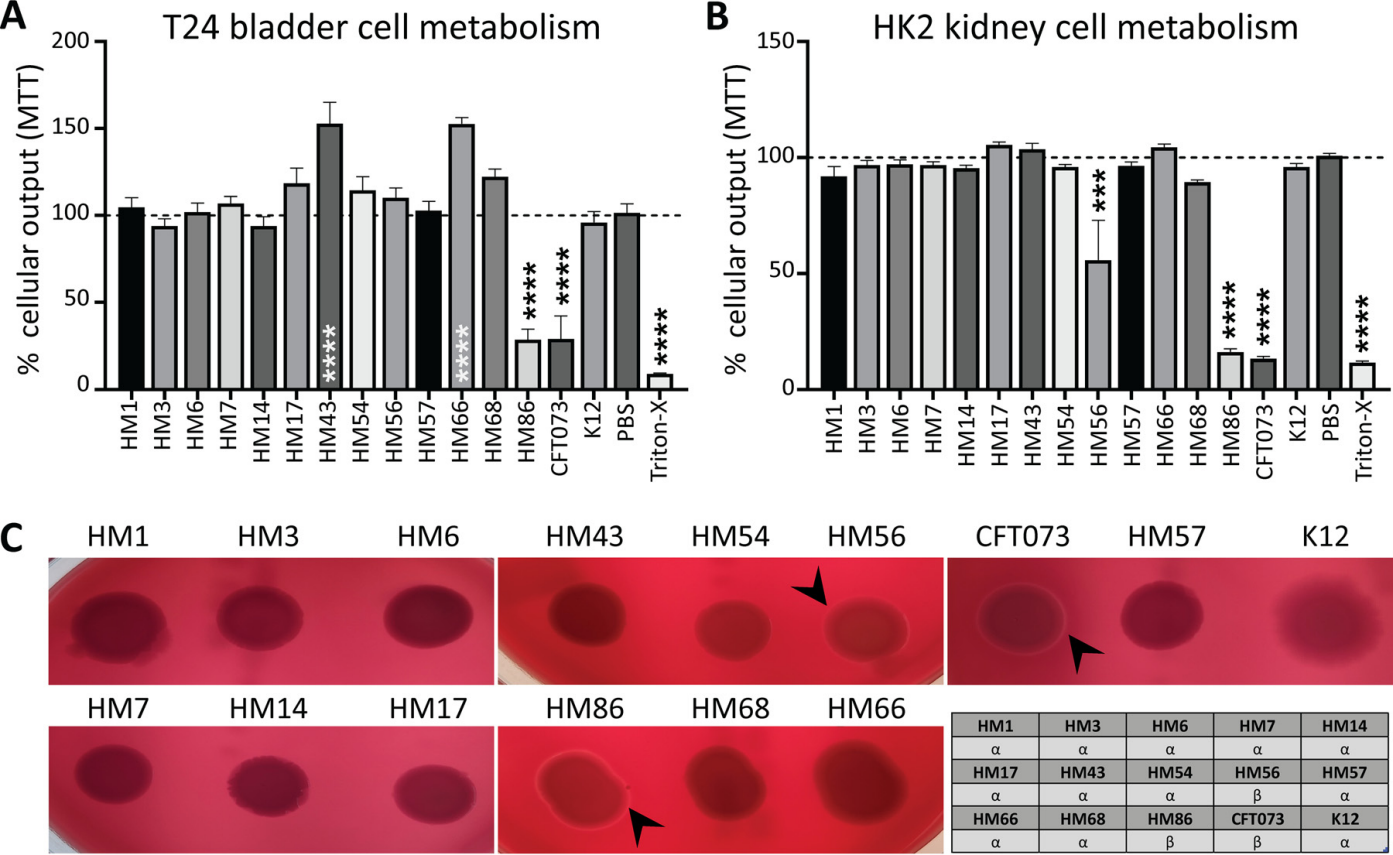
HM56 and HM86 uniquely exhibit alpha-hemolysis and can kill uroepithelial cells in culture. (A and B) To determine the ability of clinical UPEC strains to lyse epithelial cells, monolayers of T24 bladder (A) or HK2 kidney (B) cell lines were treated with UPEC for 5 h. The MTT cell viability kit was used to measure monolayer viability pre- and posttreatment with UPEC. Cellular respiration causes a color change, which can be measured via absorbance. These data were normalized, and mean values of the results of four biological replicates are represented by bars, with error bars indicating the SEM. RPMI cell culture medium with no bacteria (PBS mock) served as a negative control, and 0.4% Triton X-100 served as a positive control for cell death. One-way ANOVA was performed using Dunnett’s multiple-test correction in comparison to PBS mock infection. ***, *P < *0.005; ****, *P < *0.0001. (C) UPEC strains were spotted onto blood agar plates and incubated overnight at 37°C. The lysis of red blood cells leads to a zone of clearance (black arrowheads) around isolated bacterial colonies. CFT073 was used as a positive control for hemolysis. The chart describes the type of hemolysis detected by each strain. Alpha-hemolysis is partial hemolysis indicated by an opaque zone. Beta-hemolysis is completed hemolysis indicated by a transparent zone.

The ability of UPEC to kill host cells is initiated by a variety of known virulence factors. For example, hemolysin and other secreted toxins are known to disrupt host cell viability and cause dysregulation to essential host cell signaling cascades (53, 54). To investigate the effect of clinical UPEC on host cell viability, we measured their interactions with bladder and kidney cell lines (Fig. 4A and B). We used a colorimetric 3-(4,5-dimethylthiazol-2-yl)-2,5-diphenyltetrazolium bromide (MTT) assay which determines the metabolic activity of cells through a colored indicator. Monolayers of host cells were treated at an MOI of 50 for 5 h before treatment with antibiotics to kill bacteria, and then host cell metabolic activity was determined. CFT073 and HM86 were the only strains that caused a statistically significant decrease in T24 bladder cell metabolic output (Fig. 4A). HM43 and HM66 seemed to have increased cell metabolic activity, but upon further investigation, we observed larger biofilm-like bacterial populations that appeared to be metabolically active even after antibiotic treatment. Since MTT can be reduced by any living organism, we had background levels in our assay from the metabolic output contributions of the bacteria.

We observed that HM86, CFT073, and HM56 were able to significantly reduce the metabolic activity of HK2 kidney cells in culture (Fig. 4B). It was interesting that the same strains displaying beta-hemolysis (transparent zone indicated by black arrowheads) on blood agar were able to affect the epithelial cell lines (Fig. 4C). The strength of the phenotype, indicated by the thickness of the clear zone, also correlated directly with the ability of HM56, HM86, and CFT073 to reduce the metabolic output of HK2 kidney cells (Fig. 4B). The rest of the E. coli strains appear to cause incomplete lysis of red blood cells (alpha-hemolysis), which presents as a diffuse halo. Other UPEC-associated toxins such as Vat, Sat, Pic, Tsh, and CNF1 were present in only a few strains, and their presence did not correlate with observed decreases in host cell metabolic output.

### Assessment of stress response phenotypes reveals diverse strain responses.

**FIG 5:**
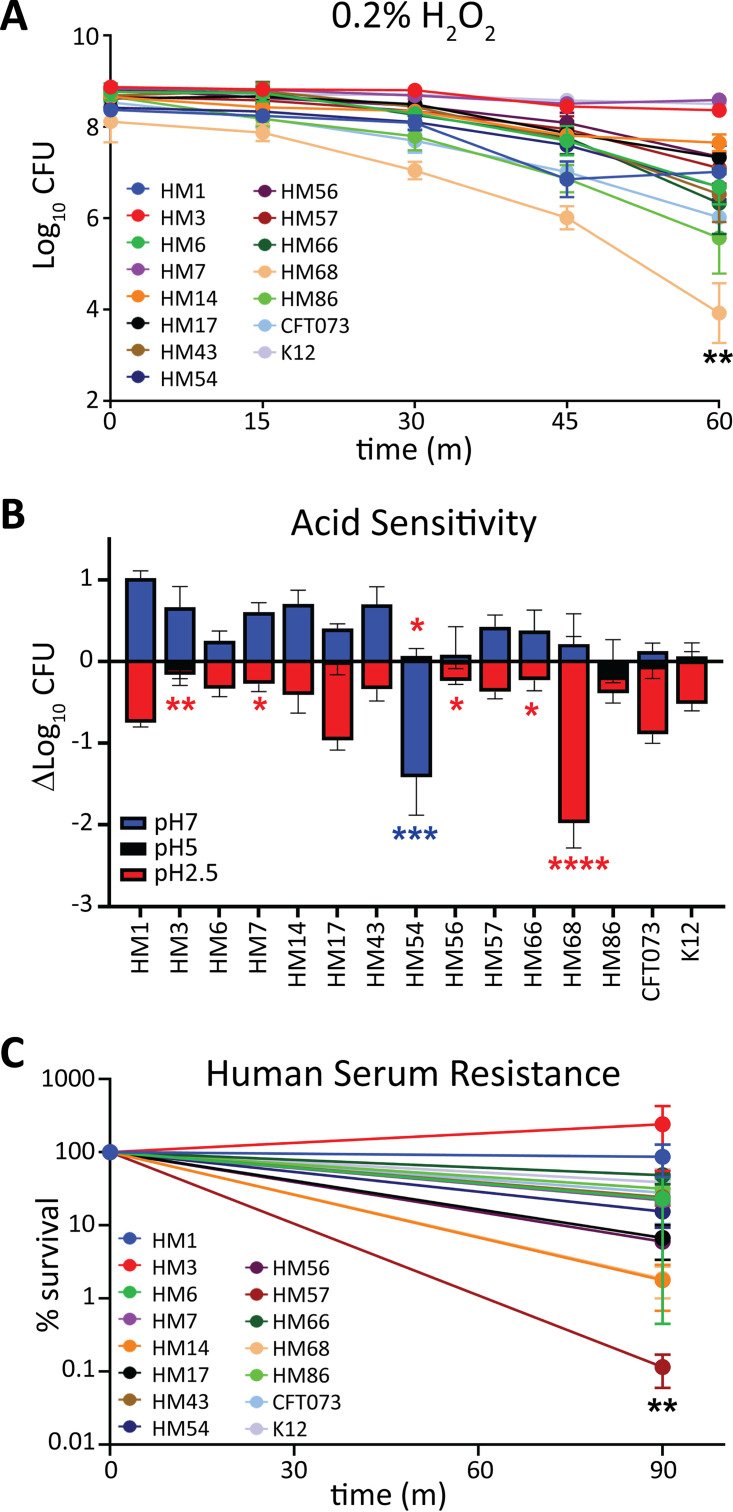
HM68 has decreased resistance to hydrogen peroxide and acid stress, while HM57 displays deficiency only in human serum resistance. (A) A total of 10^8^ CFU/mL of UPEC were inoculated into LB containing 0.2% H_2_O_2_ and maintained statically at room temperature. Samples were taken and serially diluted at 0, 15, 30, 45, and 60 min to enumerate the CFU/mL. The means of results of four biological replicates with the associated SEM are shown. (B) From an overnight culture, UPEC isolates were diluted to 10^8^ CFU/mL in LB buffered with MES to pH 7 (blue), 5 (black), or 2.5 (red). Cultures were incubated for 2 h at 37°C with aeration, and the CFU/mL were quantified at both 0 and 2 h to calculate the change in CFU/mL. Error bars indicate the SEM calculated from the mean result of four biological replicates. (C) UPEC isolates were cultured overnight in LB and washed and resuspended in PBS, and then 10^8^ CFU/mL was added to 100% complete human serum. Samples were incubated statically at 37°C for 90 min, and the percent survival (output/input) of samples was enumerated and plotted. Bars represent the mean result of four biological replicates with the SEM indicated by error bars. One-way ANOVA was performed with Dunnett’s multiple-test correction in comparison to CFT073. *, *P < *0.05; **, *P < *0.01; ***, *P < *0.005; ****, *P < *0.0001.

When in the urinary tract, bacteria are exposed to a variety of host-mediated stress responses, including osmotic stress, pH changes, bombardment from the immune system, and nutrient limitation. It is plausible that pathogens may have enhanced responses to these environmental stressors. Neutrophil infiltration is one of the host’s first lines of innate defense against UTI (55). Neutrophils attack bacteria by releasing reactive oxygen species (ROS) contained in their granules. Survival in H_2_O_2_ is designed to assess the viability of bacteria in the presence of extracellular ROS. A kill curve for each strain was determined by plotting the mean CFU/mL every 15 min for 1 h in 0.2% H_2_O_2_ (Fig. 5A). Three strains (HM3, HM7, and K-12) were the most resistant to H_2_O_2_ (Fig. 5A). HM68 was the most highly affected strain, with a 10,000-fold loss of CFU over 60 min (Fig. 5A). Increased susceptibility to death via extracellular ROS might suggest a lower *in vivo* survival rate during infection; however, shockingly, commensal isolate K-12 was one of the least susceptible strains (8-fold reduction in CFU), indicating that this phenotype may not be predictive or all encompassing of the immune interaction experienced during UTI.

We then performed a series of assays to investigate pH-induced stress responses. We examined UPEC growth and survival after a 1-h exposure to LB buffered to pH 7, 5, and 2.5 to cover the range of both the gut, urine, and neutrophil attack that UPEC may encounter in the host. (Fig. 5B). We observed that most strains had growth up to 10-fold or had no change in CFU at pH 7. The exception was HM54, which experienced over a 14-fold decline in population (Fig. 5B). Interestingly, this strain was one of the most tolerant to pH 2.5, along with HM3 (Fig. 5B). Most strains experienced a 10-fold or less reduction in CFU/mL in acidic medium except for HM68, which had a 20-fold loss (Fig. 5B). Under these conditions, the results for E. coli K-12 were not different from the average result of UPEC strains.

Complement-mediated killing is also an important innate immune defense that can be tested by measuring bacterial survival in human serum. We treated the strains with 100% human serum for 90 min and enumerated the CFU. Only a single strain, HM57, was susceptible to serum (Fig. 5C), with a loss of about 10^4^ CFU compared to input. This loss of CFU was not observed when HM57 was incubated with heat-inactivated serum (Fig. S4), indicating that the killing was complement mediated. K-12 was comparably resistant to serum, a surprising result given that it is a commensal isolate.

10.1128/msystems.00827-22.4 FIG S4 The indicated strains were cultured overnight in LB, washed in PBS, diluted to 10^8^ CFU/mL, and incubated in heat-inactivated human serum for 90 min at 37°C. Input is enumerated in the black bars, while output is enumerated in the grey bars. Bars represent the average result of four biological replicates, and error bars indicate the SEM. Download FIG S4, TIF file, 0.3 MB. Copyright © 2022 Shea et al. 2022 Shea et al. https://creativecommons.org/licenses/by/4.0/ This content is distributed under the terms of the Creative Commons Attribution 4.0 International license.

### Murine model of ascending UTI was used to assess strain infectivity.

**FIG 6:**
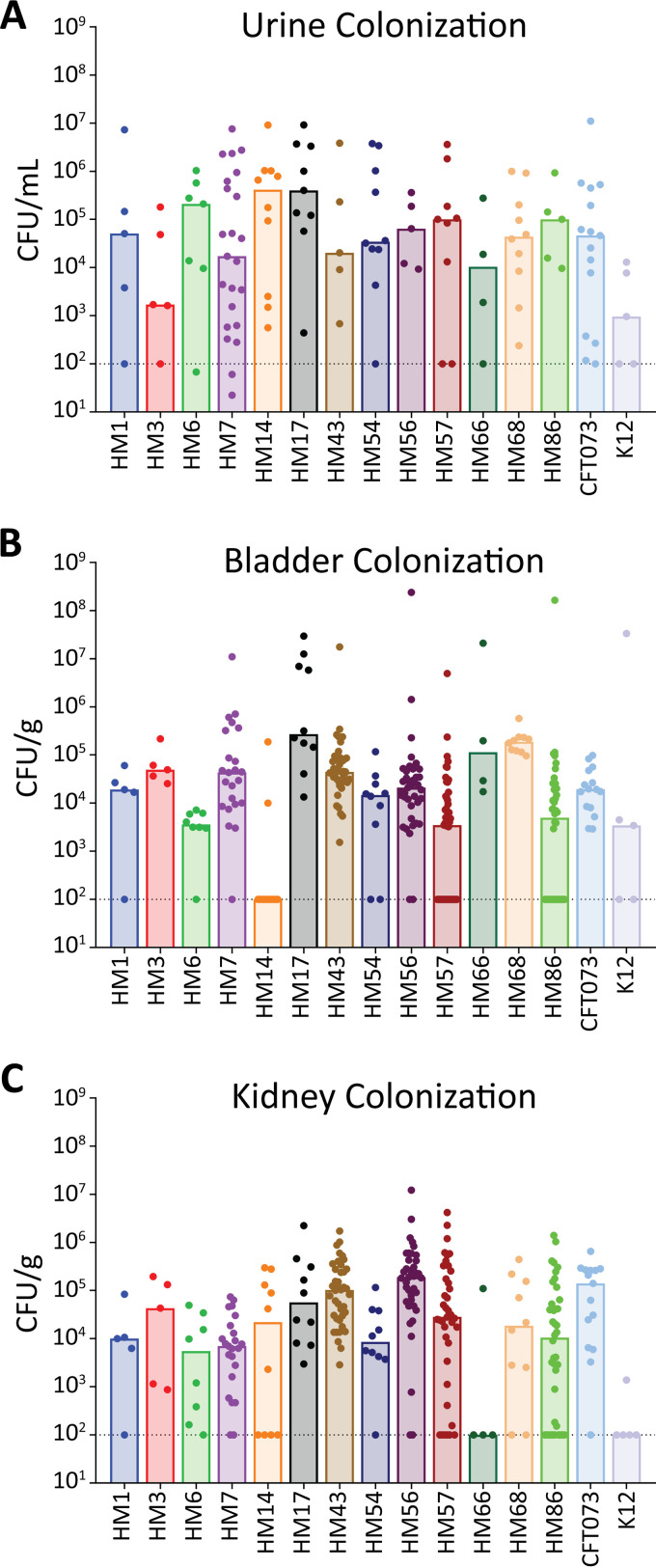
Human UPEC isolates readily establish UTI in mice. Six- to eight-week-old CBA/J mice were transurethrally inoculated with 10^8^ CFU of UPEC. The infection progressed for 48 h before urine, bladder, and kidneys were harvested to quantify the bacterial burden in each organ site. The limit of detection is 100 CFU per g of tissue or per mL of urine and is indicated by a dashed line; bars denote the median bacterial burdens. Each strain was tested on 5 to 40 CBA/J mice, and dots represent individual mice. Results for HM43, HM56, and HM86 were previously published (33, 36). (A) Urine samples were collected from each mouse and brought up to a final volume of 150 μL in sterile PBS. The number of CFU/mL of urine was determined for each individual mouse. (B and C) Bladder (B) and kidneys (C) were aseptically harvested, homogenized in 3 mL of sterile PBS, and then plated on LB agar to determine the number of CFU/g of tissue.

While these strains were isolated from women with symptomatic UTI, making them pathogenic (32, 33), we had not yet tested their ability to establish disease in our mouse model of UTI. We inoculated animals with each of the clinical isolates as well as CFT073 and K-12 and used the colonization data of HM43, HM56, and HM86 from a previous study (36). At 48 h postinoculation, we enumerated the bacterial burden in the urine, bladder, kidneys, and spleen (Fig. 6; Fig. S5). Each strain was able to colonize at least one site, and most strains were able to colonize to levels similar to that of CFT073 (Fig. 6). However, some strains displayed a preference for specific organ sites. Overall, the spleen was the least-colonized organ site, in which only seven strains were able to achieve detectable CFU (Fig. S5). We were unable to recover CFU from the bladders of mice infected with HM14, but both the urine and kidneys of these mice were highly colonized (Fig. 6). This observation is especially interesting given that HM14 does not encode type 1 fimbriae, which mediate binding to bladder epithelium (56). In contrast, HM66 robustly colonized the urine and bladders of mice but was absent in the kidneys. The differences in colonization between these strains, and specifically the preference for particular organs, could be illuminating in dissecting the virulence factors essential to these tissue-specific advantages.

10.1128/msystems.00827-22.5 FIG S5 Bacterial burden in the spleen of mice transurethrally inoculated with the indicated strains. Mice were inoculated with 10^8^ CFU, and infection progressed for 48 h. Spleens were aseptically removed and homogenized, and the bacterial burden was enumerated. Bars represent the median, and dots represent individual animals. Download FIG S5, TIF file, 1.9 MB. Copyright © 2022 Shea et al. 2022 Shea et al. https://creativecommons.org/licenses/by/4.0/ This content is distributed under the terms of the Creative Commons Attribution 4.0 International license.

### Mathematical modeling of bacterial burden using phenotypic data to develop predictors of UPEC and UTI severity.

Finally, we wanted to create a holistic model to predict infectivity using this wealth of phenotypic data. We used multiple linear regression to predict the bacterial burden in the mouse model, which acts as the proxy for infectivity.

However, this model is only predictive of normally distributed data. Therefore, using the Shapiro-Wilk test, we determined whether the urine, bladder, and kidney colonization data were normally distributed. We found that only the bladder and urine data were normally distributed (Fig. S6A), so unfortunately, we could not use this model to predict kidney colonization. Another stipulation when multiple linear regression is used is that none of the input factors can be correlated with one another. Therefore, we created a correlation matrix between all 18 of the phenotypic assays we performed (Fig. S6B), with a cutoff *R*^2^ value of 0.5 as “correlated.” We found that all growth conditions in M9 correlated with one another, as did acid sensitivity. Growth in urine correlated with all M9 growth conditions, except when glucose was the sole carbon source. We also observed expected correlations; the percentage of the bacterial population with *fim* invertible element in the on position correlated with both hemagglutination and motility. 

10.1128/msystems.00827-22.6 FIG S6 Pearson correlation matrix of all 18 phenotype assays. Values used for each assay are shown in Table S2 in the supplemental material. Asterisks denote *R*^2^ values greater than 0.5. Download FIG S6, TIF file, 0.6 MB. Copyright © 2022 Shea et al. 2022 Shea et al. https://creativecommons.org/licenses/by/4.0/ This content is distributed under the terms of the Creative Commons Attribution 4.0 International license.

**FIG 7:**
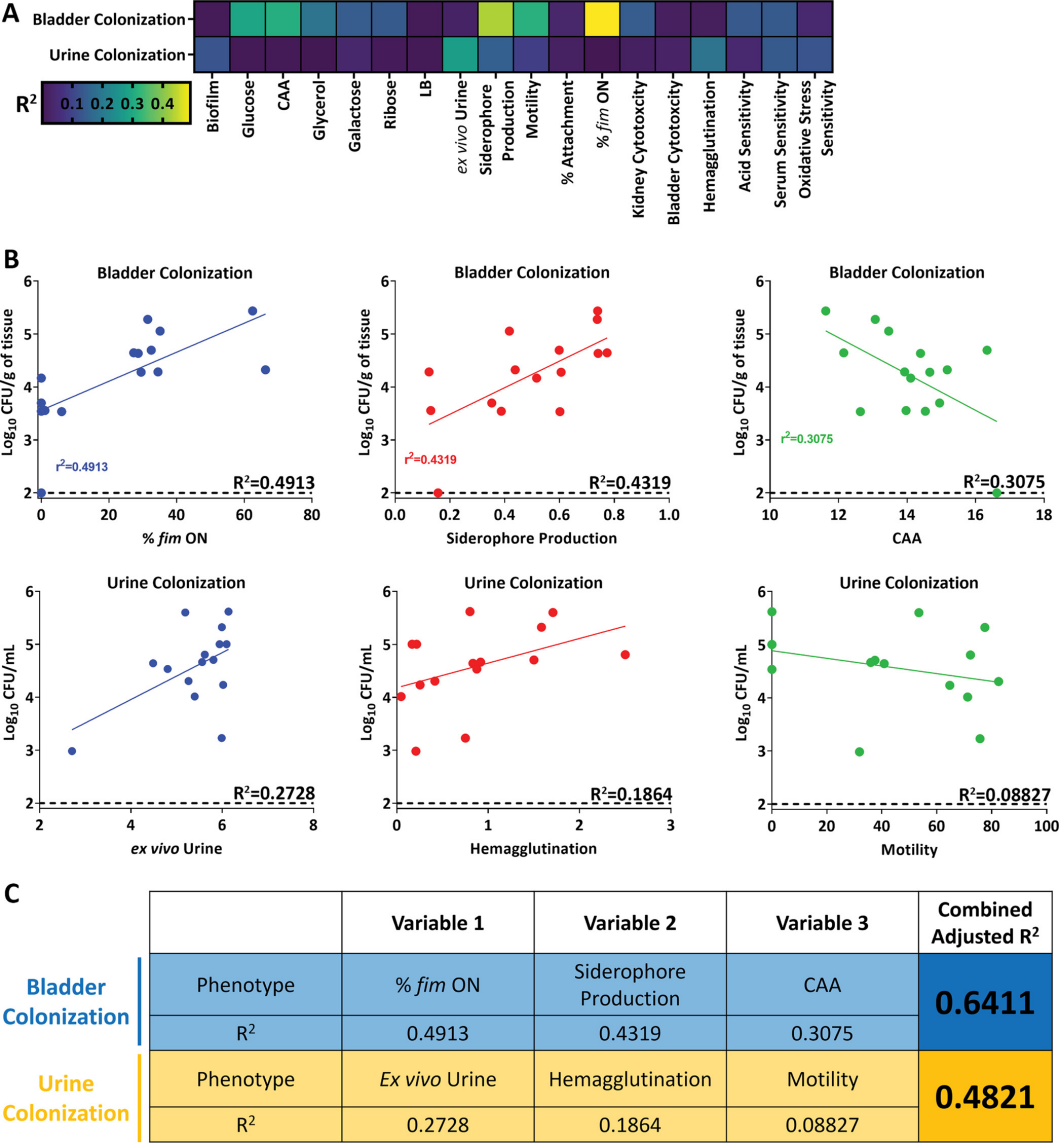
Multiple linear regression can partially predict levels of bladder and urine colonization. (A) Pearson’s correlation between either bladder colonization or urine colonization for each indicated phenotype assay. (B) Select phenotypes used in multiple linear regression with displayed *R*^2^ values based on Pearson’s correlation. (C) Multiple linear regression used to model either bladder or urine colonization. Each variable used in the model is displayed with its corresponding single *R*^2^ value, as well as the final adjusted *R*^2^ value when all three variables are taken into account.

From there, we correlated each of these phenotypic assays with either urine or bladder colonization (Fig. 7A; Table S1). Some factors were clearly strongly correlated with colonization. For example, type 1 fimbrial expression was highly correlated with bladder colonization, and growth in *ex vivo* urine correlated with bacterial burden in urine during experimental UTI. Guided by these single regressions, as well as manual curation based on previous studies, we used type 1 fimbrial expression, siderophore production, and growth in M9 with CAA as the variables for bladder colonization (Fig. 7B). The adjusted *R*^2^ for the multiple linear regression model was 0.6411, compared to the single *R*^2^ values of 0.4913, 0.4319, and 0.3075 used for type 1 fimbria expression, siderophore production, and growth in M9 with CAA, respectively (Fig. 7C). For urine colonization, we used growth in *ex vivo* urine, hemagglutination, and motility (Fig. 7B). The adjusted *R*^2^ for this combined model is 0.4821, a robust increase from 0.2728, 0.1864, and 0.08827 for *ex vivo* urine, hemagglutination, and motility, respectively (Fig. 7C). Together, these findings underscore the wide diversity of virulence strategies that UPEC can utilize and how this diversity might drive a corresponding difference in tissue tropism and fitness.

10.1128/msystems.00827-22.7 TABLE S1 Pearson’s correlation coefficient for each phenotype assay with murine urine and bladder colonization data. Download Table S1, DOCX file, 0.01 MB. Copyright © 2022 Shea et al. 2022 Shea et al. https://creativecommons.org/licenses/by/4.0/ This content is distributed under the terms of the Creative Commons Attribution 4.0 International license.

## DISCUSSION

UPEC isolates are an incredibly diverse group of pathogens among an already extremely varied species (57, 58). This population causes a variety of disease pathologies, including uncomplicated, complicated (e.g., catheter-associated), asymptomatic, and recurrent urinary tract infections. Accordingly, UPEC isolates have a large accessory genome where most uropathogen-specific genes reside. Interestingly, we have found that some of our clinical isolates do not even possess the well-established genes that are generally considered to be essential for UPEC pathogenesis, such as those encoding type 1 fimbriae (40), though the redundancy of the UPEC genome could compensate for the lack of these virulence factors. Alternatively, these clinical isolates lacking traditional virulence factors could be encoding novel proteins absent within UPEC prototype strains. To understand the virulence strategies of these uncharacterized strains, we decided to take a functional approach rather than a genetic one, since essential virulence mechanisms often have corresponding phenotypes. We hypothesized that using phenotypic assays, as opposed to genotyping, would be more revealing of the uropathogenic potential of these strains.

Therefore, we performed an extensive series of phenotypic microbiological assays to quantitatively describe bacterial urovirulence traits common to UPEC strains. We then used linear regression to correlate phenotype outcomes to bacterial burden in an ascending model of murine UTI to quantify disease severity. We found that growth in minimal medium with CAA as a sole carbon source, siderophore production, and type 1 fimbrial expression were able to best predict bladder colonization outcomes. Urine outcomes were best modeled by bacterial motility, hemagglutination, and growth in human urine *ex vivo*. Unfortunately, although we determined the bacterial burden in the kidneys and spleen (indicating bacteremia), we were unable to perform this correlation analysis since the data were not normally distributed.

Our data suggest that growth in minimal medium with CAA as a sole carbon source is a good indicator of bladder colonization, not to mention that all tested UPEC strains were able to cause either complete or incomplete lysis of red blood cells. It is conceivable that this cellular damage further encourages the degradation of host cells leading the release of protein into the extracellular milieu. However, the correlation between bladder colonization and growth in CAA was negative, even though UPEC isolates prefer amino acids to carbohydrates as carbon sources during infection (37, 59). However, CAA are a mix of single amino acids, and the nutrient milieu in the infected bladder is far more likely to contain oligopeptides or even whole proteins from lysed cells. Indeed, several di- or tripeptide transporters are virulence factors in the mouse model (37). To use CAA as a carbon source, UPEC would need to rely on the upregulation of single amino acid transporters instead of less-specific di- or tripeptide transporters as observed *in vivo*.

We also observed that three clinical strains, HM17, HM43, and HM68, had restricted growth with sole carbohydrate sources in minimal medium. However, these strains had some of the highest bacterial burdens in the bladders of mice, showing that sugar utilization is dispensable for UPEC in the infection niche, corroborating several previous studies (33, 37, 59). It is interesting to speculate that these strains might have defects in gut colonization, where sugar utilization is key (60). Conversely, perhaps commensal gut organisms have efficient sugar utilization uptake and rely less heavily on amino acid-based metabolic processes.

Iron sequestration mechanisms are highly expressed during infection (32, 33, 61) but are not part of the core genome of E. coli (33). There are four main UPEC siderophore systems used for scavenging iron in the host: yersiniabactin, aerobactin, enterobactin, and salmochelin (29, 31, 62). The clinical strains encoded between one and three of these, while CFT073 encoded three. We observed differences between strains in a liquid CAS assay, but every strain had CAS activity when assessed by the more-sensitive agar-based method. Interestingly, strains with lower liquid CAS activity had higher bladder colonization (HM17, HM43, and HM68). Siderophore production comes at a high metabolic cost, and perhaps these lower-producing strains have fine-tuned the production of these small molecules while the others are overproducing.

Type 1 fimbriae are a preeminent virulence factor for murine bladder adhesion, invasion, and colonization (63). An invertible element assay determined the orientation of the type 1 fimbrial promoter in either the on or off orientation *in vitro*, and the results were highly predictive of bladder colonization in the murine model. Correspondingly, we were unable to recover any CFU from the bladders of mice inoculated with HM14, which does not encode type 1 fimbriae. However, this strain was able to robustly colonize the urine and kidneys of the mouse. Thus, although type 1 fimbriae are critical for establishing cystitis, they are dispensable for colonization of other sites in the urinary tract. The presence of the *fim* operon is necessary but not sufficient to predict bladder colonization, as nonpathogenic K-12 isolates also possess this fimbria. However, by using the invertible element assay, we revealed that the invertible element was overwhelmingly in the off position in K-12. This would phenocopy a strain that does not encode the fimbria, demonstrating the utility of phenotypic testing over genotyping.

HM14 was particularly unique in more ways than its lack of the *fim* operon. It also had a more mucoid appearance, complete exclusion of Congo red dye, and a lack of motility. The lack of motility is particularly interesting given the strain’s high kidney colonization, considering that previous literature demonstrates the critical role of flagellum-mediated motility in ascension of the urinary tract (42). However, we must consider that *in vitro* conditions such as temperature, nutrient availability, and presence of ROS, among other factors, are different from those of the murine host. This may lead to different expression levels of flagella in the host environment.

Overall, we found that the single phenotype correlations relating back to urine colonization were weaker than the ones for bladder colonization. The strongest single correlation was a positive correlation with human urine growth *ex vivo*, a logical observation given that the infection milieu of the urinary tract is urine. However, it is worth noting that the data are skewed; K-12 has a severe defect in growth and colonization in urine, while the UPEC strains are clustered more tightly together (Fig. 7B). Interestingly, bacterial motility had a mild negative correlation with urine colonization. This might indicate that more motile strains are better able to move and associate with the bladder epithelium, ascend into the kidneys, or are more highly recognized by the TLR5-mediated host clearance mechanisms; any or all of these are possible.

One of the relatively stronger associations was a positive correlation between bacterial hemagglutination of guinea pig red blood cells and urine colonization. Likewise, a previous study found hemagglutination to predict bladder colonization in C3H/HeN mice (10). It was speculated that some of the observed differences between *in vivo* and *in vitro* conditions were likely due to differential responses to host niche-specific environmental cues (10). It is known that bladder epithelial cells are shed into the urine as a host defense mechanism to infection, so it is plausible that host cell-bound bacteria could be concentrated in the murine urine sample, also explaining the association.

Collectively, we demonstrate that prediction of virulence potential and assessment of infection severity are better determined through phenotypic analyses. The current limitations in the potential clinical implementation of these assays come from expense, time, and a lack of standardized experimental conditions that more accurately model the host. However, the use of these current phenotypic characterization assays increases the prediction efficiency of UPEC pathogenic potential. Future studies will work to improve these assays and make them more reflective of the host environment, hopefully resulting in even better predictive outcomes.

## MATERIALS AND METHODS

### Bacterial and cell culture conditions.

E. coli CFT073 was isolated from the blood and urine of a hospitalized patient with acute pyelonephritis (22). Clinical HM strains were collected from the urine of young, healthy females presenting to the University of Michigan clinic with symptomatic UTI (32, 33). Luria broth (LB), which contains, per liter, 0.5 g NaCl, 5 g yeast extract, and 10 g tryptone, was used to routinely culture bacteria at 37°C under aerated or static conditions and was inoculated from single colonies.

Human epithelial T24 bladder cells (ATCC HTB-4) and HK2 kidney cells (ATCC CRL-2190) were maintained in RPMI medium containing l-glutamine (Corning product no. 10-040-CV) with 10% fetal bovine serum (FBS; Corning product no. MT35010CV) and (10 mg/mL) penicillin/streptomycin antibiotics (Corning product no. MT30002CI) at 37°C and 5% CO_2_. All procedures involving human samples were performed in accordance with the protocol (HUM00029910) approved by the Institutional Review Board at the University of Michigan. This protocol is compliant with the guidelines established by the National Institutes of Health for research using samples derived from human subjects.

### Bacterial growth curves.

Overnight LB bacterial cultures were washed once with phosphate-buffered saline (PBS) and then inoculated 1:100 into 1 mL of LB or M9 minimal medium (64), supplemented with either 0.4% glucose, glycerol, galactose, ribose, or Casamino Acids (CAA), or into filter-sterilized pooled human urine collected from at least four female donors. These cultures were incubated with aeration at 37°C in a Bioscreen C automated growth curve analyzer (Growth Curves USA), with optical density at 600 nm (OD_600_) readings collected every 15 min for 24 h. The area under the curve (AUC) was calculated using GraphPad 9.3.1.

### Anaerobic sugar fermentation.

The anaerobic sugar fermentation protocol was adapted from that of Himpsl et al. (65). Briefly, 5 μL of overnight LB cultures of each strain was spotted onto LB agar containing phenol red (0.04 g/L) alone or supplemented with 0.4% glucose, 0.4% glycerol, 0.4% galactose, or 0.4% ribose. Plates were incubated for 24 h at 37°C under anaerobic conditions (BD GasPak EZ anaerobe) and then imaged. Plates were transitioned to aerobic conditions and incubated at room temperature for another 6 or 24 h, with imaging at each time point. In parallel, strains were also spotted on plates that were incubated under aerobic conditions at 37°C for 24 h, before transition to room temperature for another 6 and 24 h. Colors were quantified from images of representative plates. The center of each colony was selected with the eyedropper tool in Adobe Photoshop to determine its color.

### Chrome azurol S assay.

Strains were cultured overnight in LB with shaking at 37°C. The overnight cultures were harvested by centrifugation, washed with PBS, diluted 1:100 into M9 minimal medium supplemented with 0.4% glucose, and incubated overnight, with shaking at 37°C. Cultures were pelleted by centrifugation, and 100 μL of supernatants was combined with 100 μL of chrome azurol S (CAS) shuttle solution (Fluka catalog no. 216-787-0) (66) and incubated at room temperature for 30 min. Ten millimolar EDTA (Fisher catalog no. AC118432500) served as a positive control. After 30 min, the absorbance at 630 nm was recorded.

### Biofilm formation.

Strains were cultured overnight in LB, with shaking at 37°C, and diluted 1:100 into either 3 mL of fresh LB medium or *ex vivo* human urine into tissue culture-treated 24-well plates (Corning product no. CLS3527) and incubated statically at either 37°C or 30°C for 24 h. The medium was carefully decanted and rinsed with distilled water to remove any nonadherent bacteria and then stained with a 0.1% solution of crystal violet for 15 min at room temperature. The stain was aspirated, and the wells were rinsed with distilled water. Biofilms were solubilized with 1 mL of 100% ethanol for 15 min at room temperature with shaking. Two hundred microliters of solubilized biofilm was read in a 96-well plate at an absorbance of 570 nm. Congo red agar was made as previously described (67). Overnight cultures (5 μL) were spotted onto Congo red agar and incubated at 30°C for 48 h before images were taken.

### Motility.

Overnight LB cultures of each strain were normalized to an OD_600_ of 1.0 and resuspended in HEPES buffer, pH 8.4. Cultures were stabbed in duplicate into tryptone agar plates (1% tryptone, 0.5% NaCl) containing 0.25% agar with an inoculating needle. Bacteria were allowed to swim for 16 h at 30°C, and then the diameter of the motility zone was measured.

### *fimS* invertible element orientation.

Bacteria were cultured overnight in LB medium statically at 37°C. The OD_600_ of cultures was taken, strains were normalized to an OD_600_ of 0.5 in water, and PCR product digestion was done as previously described (51). The entire sample was run on a 3% agarose gel at 100 V to visualize *fimS* on and off orientation bands. A representative gel is shown in Fig. S3A in the supplemental material. The intensity of the bands was quantified using Bio-Rad software.

### Hemagglutination.

Bacteria were cultured statically in LB at 37°C for 72 h (55). Cells were washed and resuspended in 1× PBS and then serially diluted 1:2 in a round-bottom 96-well plate. Guinea pig erythrocytes (Innovative Research catalog no. IGPRBC10ML-33782) were washed, resuspended at 3% in PBS (vol/vol), and then added to each well of the plate. Bacteria and erythrocytes were gently mixed and allowed to settle for 1 h. To assess the role of the type 1 fimbriae, 50 mM mannose was added to interfere with fimbrial binding as previously described (59).

### Cell association.

Cell lines were cultured under conditions previously described to confluence in 24-well plates (Corning) and serum starved overnight before adhesion assays were performed. Bacteria were cultured overnight in static LB at 37°C. Cells were trypsinized (Corning catalog no. 25-053-CI) and counted using a hemacytometer, and the number of bacterial CFU/mL of overnight cultures was determined via OD_600_. An MOI of 1:100 was used for these assays, with bacteria normalized in 1 mL serum-free RPMI medium and added to epithelial cell monolayers for 1 h of incubation. Medium containing bacteria was aspirated, and cell monolayers were gently washed with 1× PBS three times with mild agitation. A PBS solution with 0.4% Triton X-100 was applied for 30 min with strong agitation to lyse epithelial cells. The resulting lysates were serially diluted and plated on LB agar to enumerate the CFU/mL of cell-associated UPEC.

### MTT cell metabolic output.

Cell viability after coincubation with E. coli was determined by utilizing a cell proliferation kit I (Millipore Sigma catalog no. 11465007001). A suspension with an MOI of 50 of each strain was added to a confluent monolayer of host cells (T24 ATCC HTB-4 or HK2 ATCC CRL-2190) and incubated at 37°C with 5% CO_2_ for 5 h. The medium was then replaced with RPMI medium containing penicillin (100 μg/mL), streptomycin (100 μg/mL), and gentamicin (100 μg/mL) and then incubated further for 2 h in an attempt to halt bacterial metabolic contributions. Monolayers were washed, and 100 μL of MTT-containing RPMI medium was added to each well, according to the kit protocol. After 2 to 4 h of incubation, 100 μL of solubilization reagent was added, and ultimately, the *A*_570_ was measured to determine cellular respiration. RPMI medium mock treated with PBS (no bacterial cells) served as the negative control, and RPMI medium with 0.4% Triton-X served as the positive control for cell metabolic output.

### Hemolysis.

Strains were cultured overnight in LB with aeration at 37°C. Overnight cultures were struck out for single colonies, or spotted, on tryptic soy agar containing 5% sheep blood (Thermo-Fisher catalog no. R01198) and incubated overnight at 37°C. Plates were observed for zones of hemolysis surrounding the colonies.

### Hydrogen peroxide and acid resistance assays.

Overnight bacterial cultures were incubated in LB medium at 37°C. The OD_600_ was taken, and cultures were normalized to 10^8^ CFU/mL in 3 mL of either plain LB or LB containing fresh 0.2% H_2_O_2_ (Fisher Scientific catalog no. BP2633500). Samples were immediately vortexed and serially diluted in PBS to enumerate the CFU/mL at time zero via drip-plating onto LB agar. Samples were incubated on the benchtop and then vortexed and plated at each time point.

For acid resistance, the cultures were also normalized to 10^8^ CFU/mL in 3 mL of LB buffered with 2-(N-morpholino)ethanesulfonic acid (MES) to pH 7, 5, or 2.5, and the input CFU/mL was determined. Cultures were then incubated for 2 h at 37°C with aeration, and the final number of CFU/mL was calculated.

### Serum resistance.

Overnight LB cultures (1 mL) were pelleted by centrifugation and resuspended in 1 mL of sterile PBS. Suspensions were diluted 1:10 in sterile PBS. Ten microliters of the diluted PBS solution was added to 190 μL of either 100% human serum or 100% heat-inactivated human serum (Innovative Research catalog no. ISER10ML). The bacterial inoculum was calculated by serially diluting and drip-plating 20 μL of the bacteria-serum suspension on LB agar plates. The mix was then incubated for 90 min at 37°C, and then the number of CFU was calculated in the same manner as for the inoculum.

### Murine model of UTI.

Female CBA/J mice (6 to 8 weeks old) from Jackson Laboratories were each transurethrally inoculated with 50 μL of a bacterial suspension (2 × 10^8^ CFU/mL) of each strain tested, using a sterile polyethylene catheter (inside diameter of 0.28 mm by outside diameter of 0.61 mm) connected to an infusion pump (Harvard Apparatus) as described previously (68, 69). The procedure was performed under anesthesia with ketamine/xylazine. Forty-eight hours postinoculation, urine was collected and then the mice were euthanized. Bladders, kidneys, and spleens were aseptically removed and homogenized. Urine and organ homogenates were serially diluted and plated onto LB agar to determine the bacterial burden. All animal protocols were approved by the Institutional Animal Care and Use Committee (IACUC) at the University of Michigan Medical School (PRO00010856) and in accordance with the Office of Laboratory Animal Welfare (OLAW). All procedures involving human samples were performed in accordance with the protocol (HUM00029910) approved by the Institutional Review Board at the University of Michigan. This protocol is compliant with the guidelines established by the National Institutes of Health for research using samples derived from human subjects.

### Multiple linear regression and Pearson correlation.

The averaged values for each assay (Table S2) were loaded into RStudio (version 1.41717). To calculate multiple linear regression, the baseR function lm() was used with three selected variables. To create a correlation matrix, the baseR function cor() was used with Pearson selected as the method.

10.1128/msystems.00827-22.8 TABLE S2 Quantitative values for phenotype assay performance used for correlation analysis. Download Table S2, XLSX file, 0.01 MB. Copyright © 2022 Shea et al. 2022 Shea et al. https://creativecommons.org/licenses/by/4.0/ This content is distributed under the terms of the Creative Commons Attribution 4.0 International license.

10.1128/msystems.00827-22.9 TABLE S3 Bacterial strains used in this study. Download Table S3, DOCX file, 0.02 MB. Copyright © 2022 Shea et al. 2022 Shea et al. https://creativecommons.org/licenses/by/4.0/ This content is distributed under the terms of the Creative Commons Attribution 4.0 International license.

10.1128/msystems.00827-22.10 TABLE S4 Primers used in this study. Download Table S4, DOCX file, 0.01 MB. Copyright © 2022 Shea et al. 2022 Shea et al. https://creativecommons.org/licenses/by/4.0/ This content is distributed under the terms of the Creative Commons Attribution 4.0 International license.
