# Supplementary figures and images for: Phenotypic Assessment of Clinical Escherichia coli Isolates as an Indicator for Uropathogenic Potential
Source: mSystems. 2022 Nov 29;7(6):e00827-22. doi: 10.1128/msystems.00827-22 (PMC9765037; doi:10.1128/msystems.00827-22)

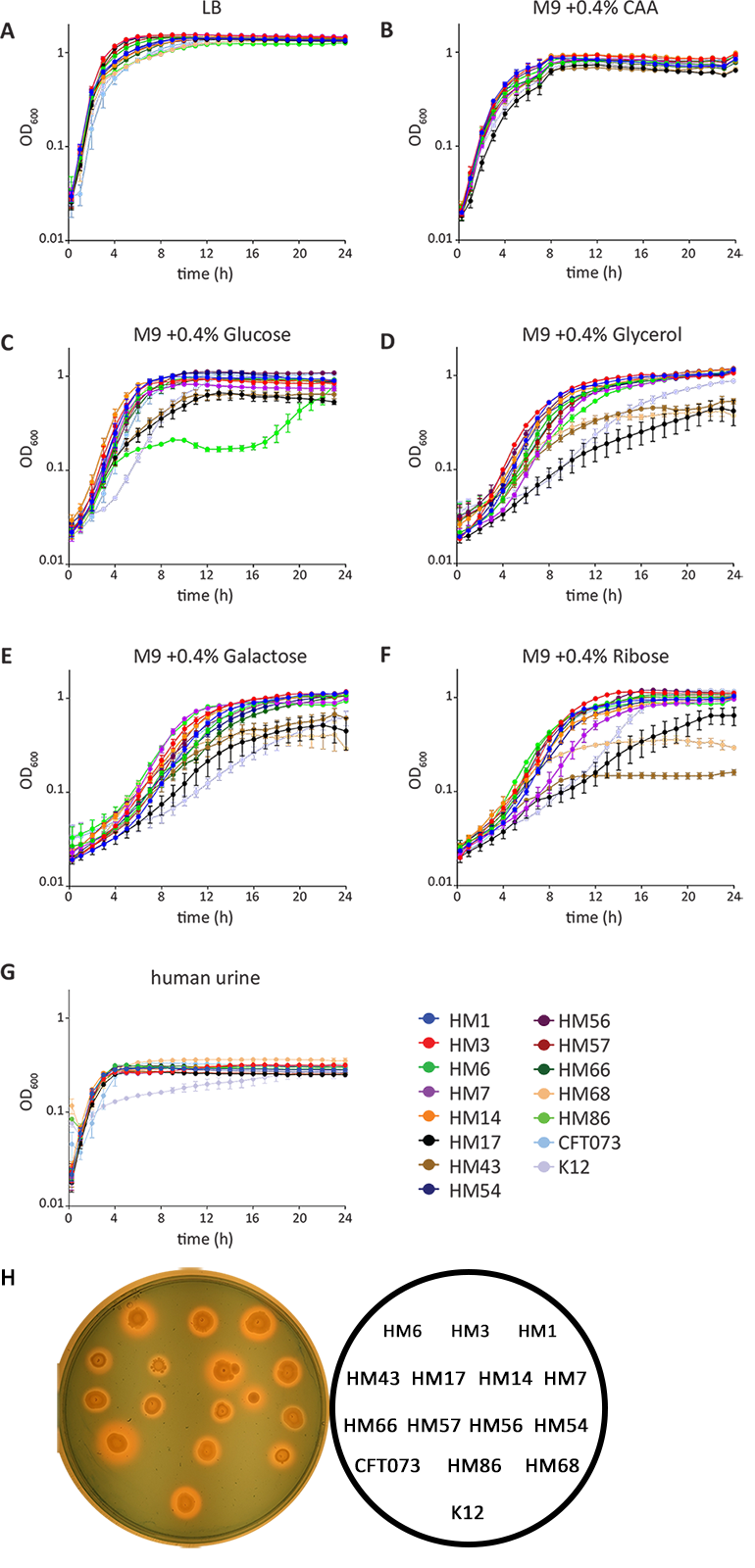

Supplement: FIG S1 [file msystems.00827-22-s0001.tif]

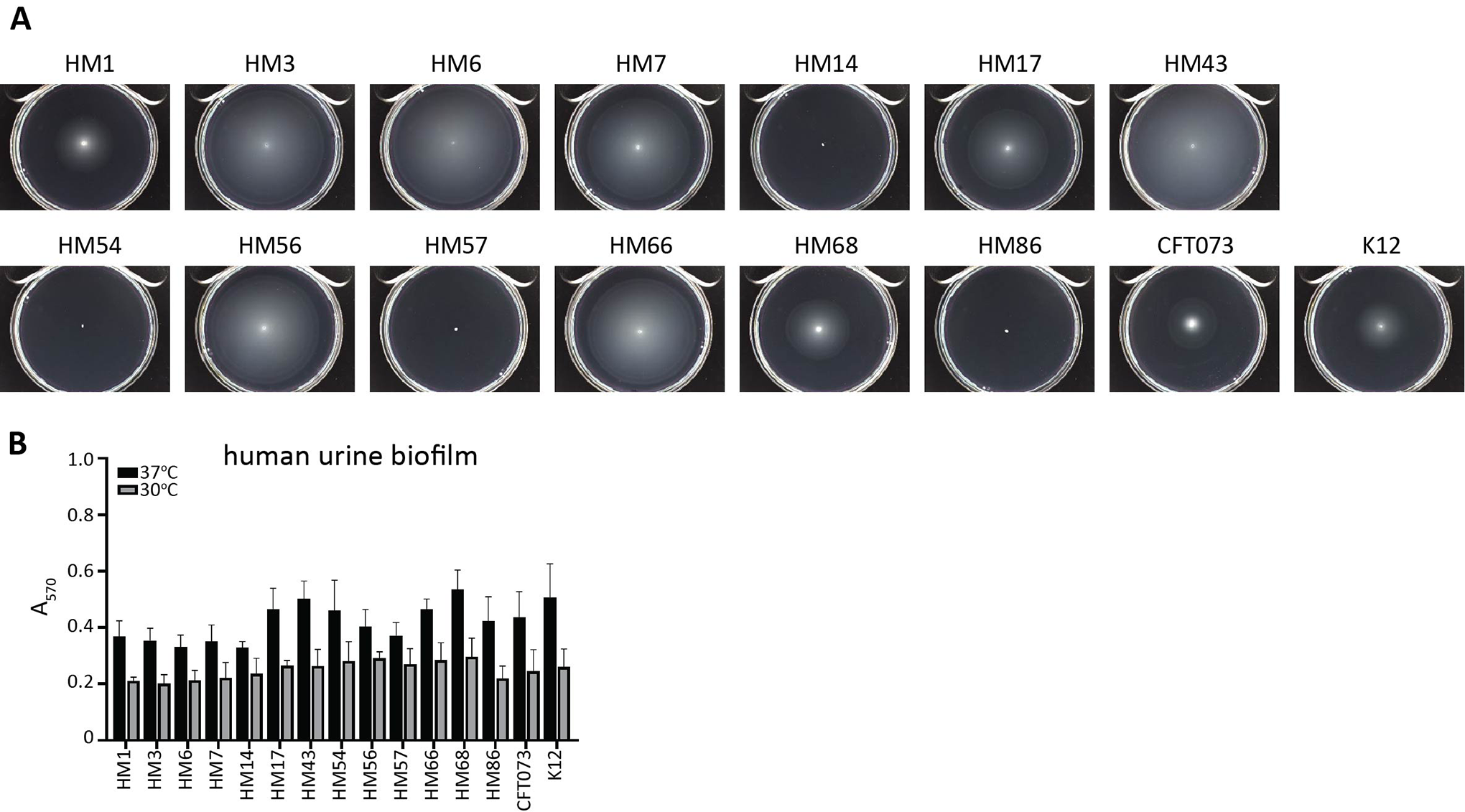

Supplement: FIG S2 [file msystems.00827-22-s0002.tif]

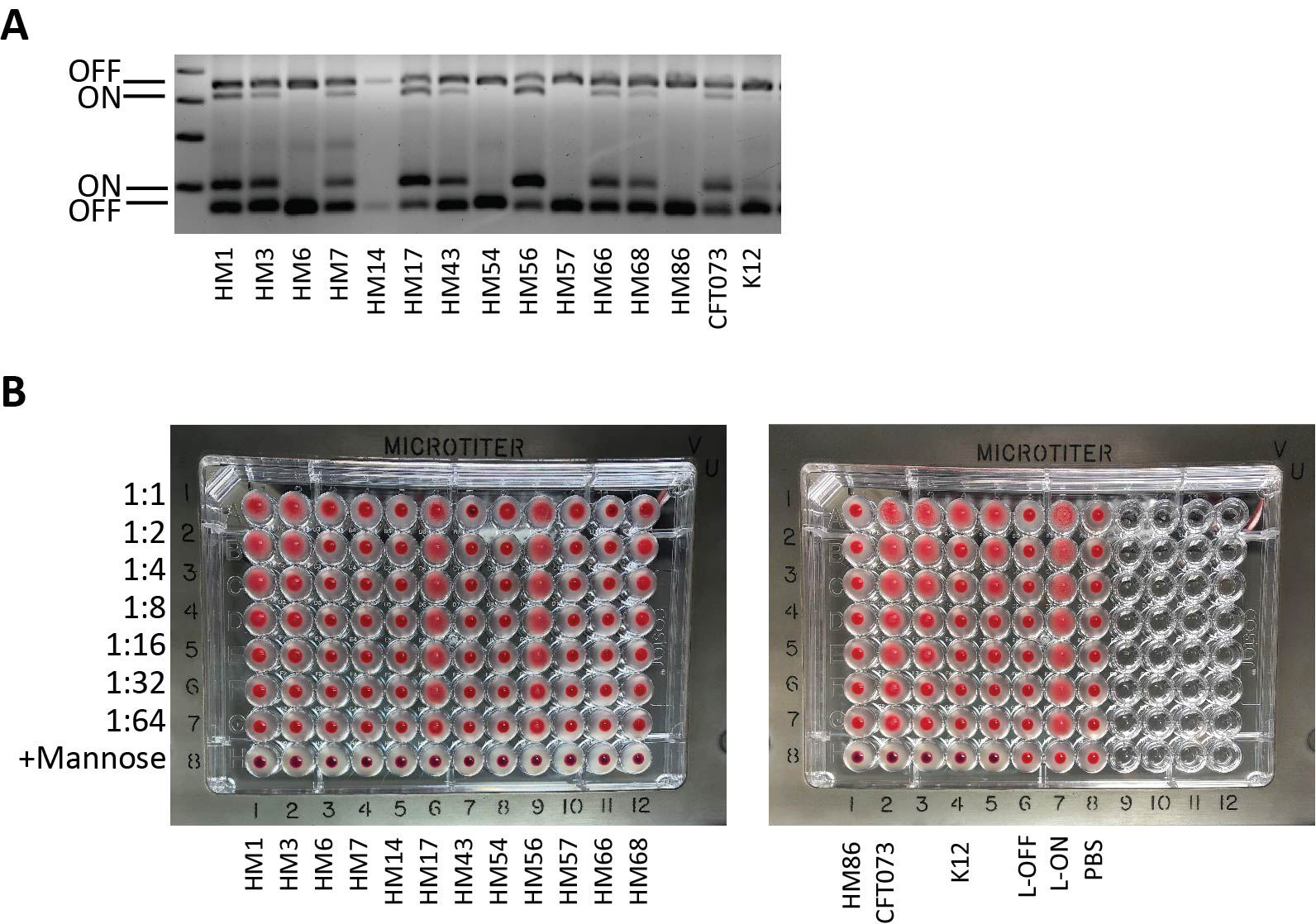

Supplement: FIG S3 [file msystems.00827-22-s0003.tif]

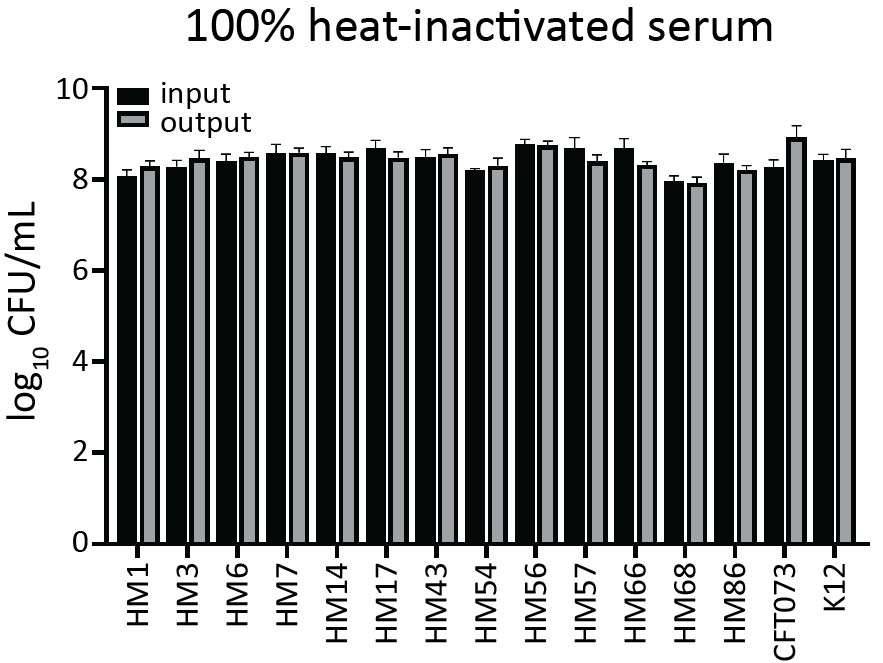

Supplement: FIG S4 [file msystems.00827-22-s0004.tif]

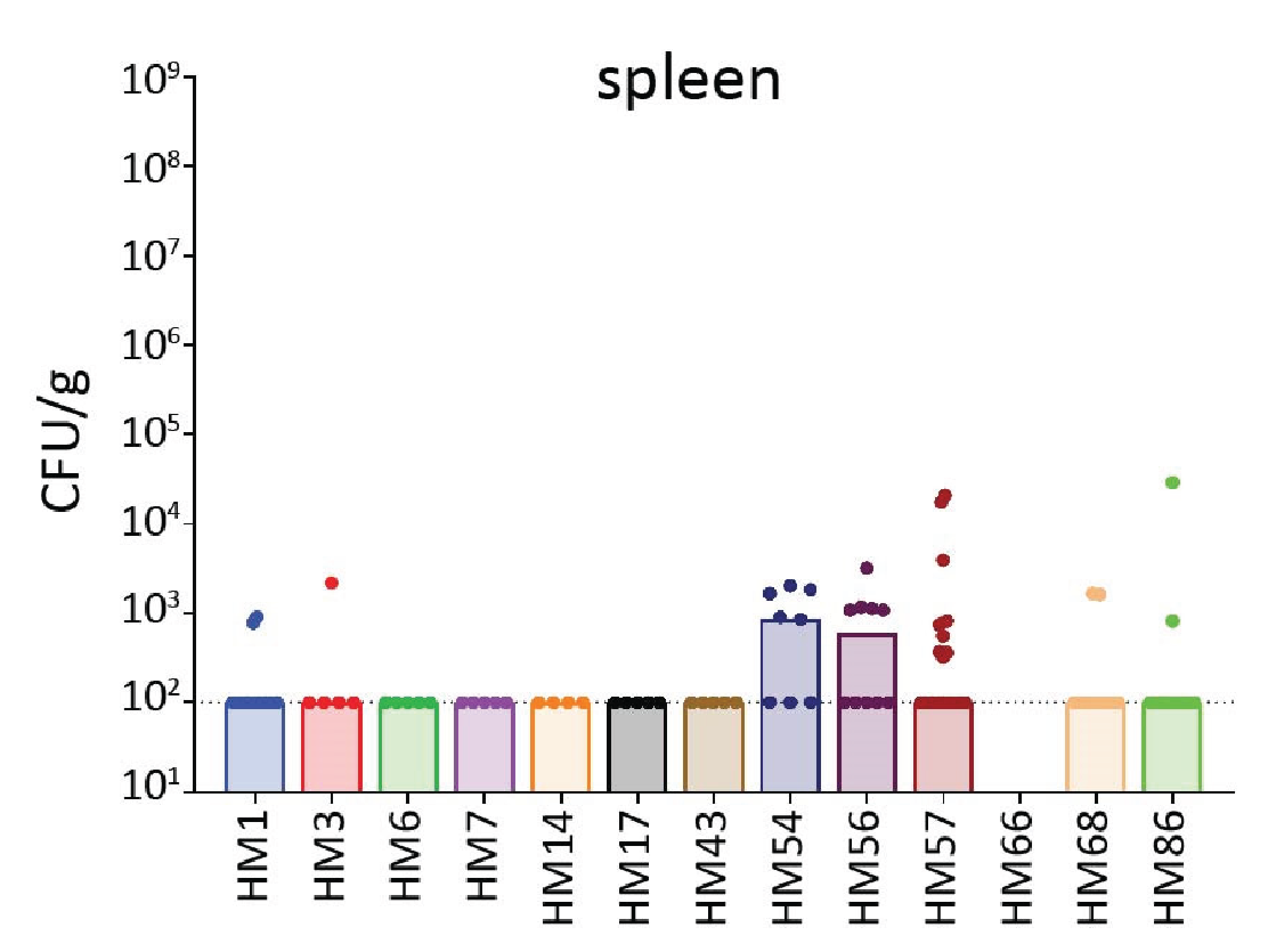

Supplement: FIG S5 [file msystems.00827-22-s0005.tif]

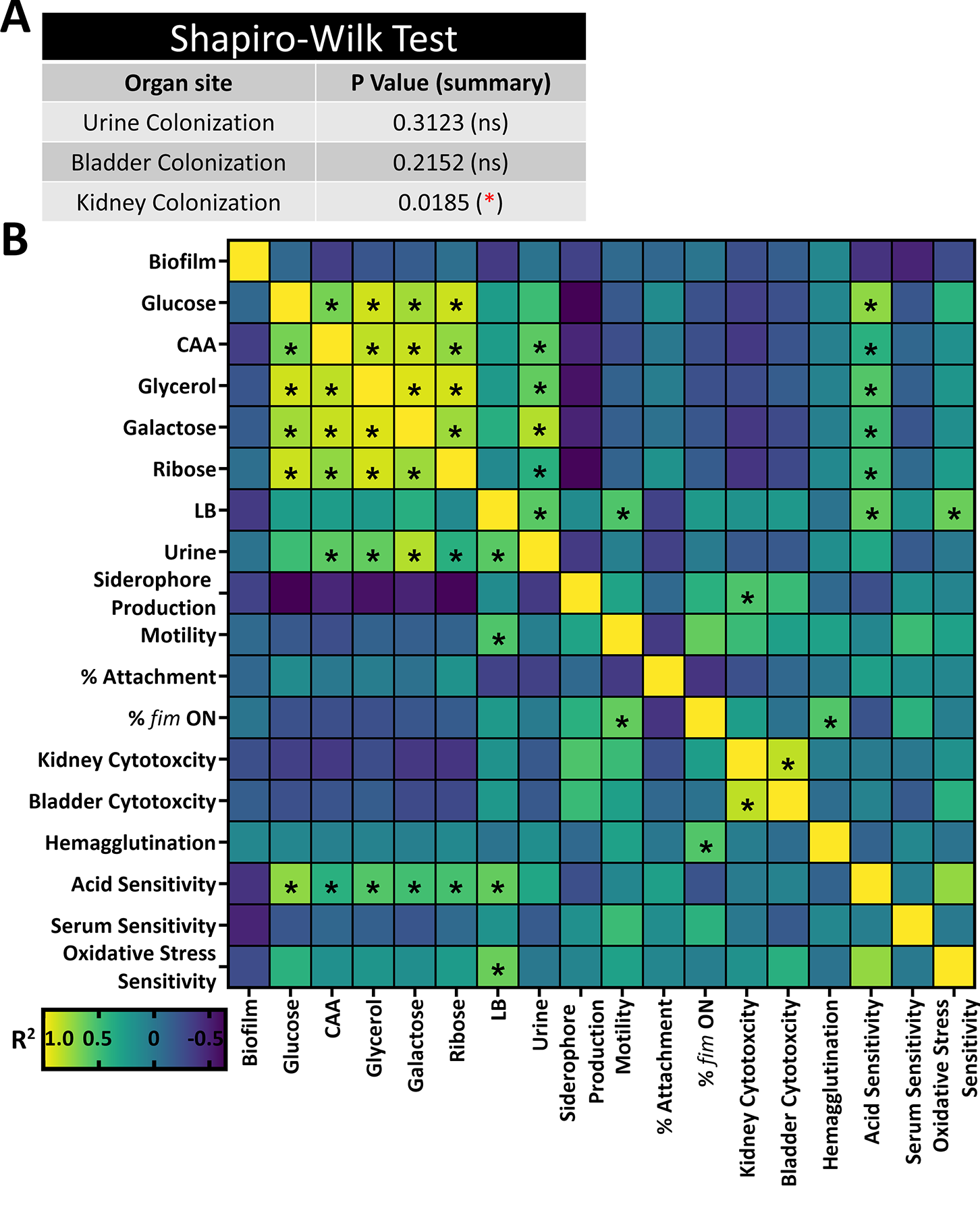

Supplement: FIG S6 [file msystems.00827-22-s0006.tif]
