# Supplementary material for: Phenotypic Assessment of Clinical Escherichia coli Isolates as an Indicator for Uropathogenic Potential
Source: mSystems. 2022 Nov 29;7(6):e00827-22. doi: 10.1128/msystems.00827-22 (PMC9765037; doi:10.1128/msystems.00827-22)
Supplement: TABLE S1 [file msystems.00827-22-s0007.docx]

|  | **Pearson’s Correlation Coefficient (R^2^)** | |
| --- | --- | --- |
| **Phenotype** | **Urine Colonization** | **Bladder Colonization** |
| Biofilm | 0.1256 | 0.004545 |
| Glucose | 0.003549 | 0.2875 |
| CAA | 0.003588 | 0.3075 |
| Glycerol | 0.0002943 | 0.1866 |
| Galactose | 0.03506 | 0.141 |
| Ribose | 0.007612 | 0.1361 |
| LB | 0.003503 | 0.004689 |
| *ex vivo* Urine | 0.2728 | 0.0213 |
| Siderophore  Production | 0.1482 | 0.4319 |
| Motility | 0.08827 | 0.3135 |
| % Attachment | 0.02056 | 0.03202 |
| % *fim* ON | 0.00005963 | 0.4913 |
| Kidney Cytotoxcity | 0.01783 | 0.1425 |
| Bladder Cytotoxcity | 0.02335 | 0.0154 |
| Hemagglutination | 0.1864 | 0.01221 |
| Acid Sensitivity | 0.03779 | 0.134 |
| Serum Sensitivity | 0.1359 | 0.1356 |
| Oxidative Stress Sensitivity | 0.1261 | 0.03974 |
