# Supplementary material for: Phenotypic Assessment of Clinical Escherichia coli Isolates as an Indicator for Uropathogenic Potential
Source: mSystems. 2022 Nov 29;7(6):e00827-22. doi: 10.1128/msystems.00827-22 (PMC9765037; doi:10.1128/msystems.00827-22)
Supplement: TABLE S3 [file msystems.00827-22-s0009.docx]

| **Strains** | **Description** | **Reference** |
| --- | --- | --- |
| CFT073 | Wild-type pyelonephritis isolate (O6:K2:H1) | (67) |
| CFT073 L-ON | CFT073 ΔIRL, *fim* invertible element locked on | (70) |
| CFT073 L-OFF | CFT073 ΔIRL, *fim* invertible element locked off | (70) |
| K12 MG1655 | Non-pathogenic laboratory strain (OR:H48:K-) | (71) |
| HM1 | Wild-type cystitis strain isolated from a healthy young woman in 2009 | (33) |
| HM3 | Wild-type cystitis strain isolated from a healthy young woman in 2009 | (33) |
| HM6 | Wild-type cystitis strain isolated from a healthy young woman in 2009 | (33) |
| HM7 | Wild-type cystitis strain isolated from a healthy young woman in 2009 | (33) |
| HM14 | Wild-type cystitis strain isolated from a healthy young woman in 2009 | (33) |
| HM17 | Wild-type cystitis strain isolated from a healthy young woman in 2009 | (33) |
| HM43 | Wild-type cystitis strain isolated from a healthy young woman in 2009 | (33) |
| HM54 | Wild-type cystitis strain isolated from a healthy young woman in 2009 | (33) |
| HM56 | Wild-type cystitis strain isolated from a healthy young woman in 2009 | (33) |
| HM57 | Wild-type cystitis strain isolated from a healthy young woman in 2009 | (33) |
| HM66 | Wild-type cystitis strain isolated from a healthy young woman in 2009 | (33) |
| HM68 | Wild-type cystitis strain isolated from a healthy young woman in 2009 | (33) |
| HM86 | Wild-type cystitis strain isolated from a healthy young woman in 2009 | (33) |
