# Supplementary material for: Phenotypic Assessment of Clinical Escherichia coli Isolates as an Indicator for Uropathogenic Potential
Source: mSystems. 2022 Nov 29;7(6):e00827-22. doi: 10.1128/msystems.00827-22 (PMC9765037; doi:10.1128/msystems.00827-22)
Supplement: TABLE S4 [file msystems.00827-22-s0010.docx]

|  | | |
| --- | --- | --- |
| Primer | 5′- 3′ sequence | |
| IE *fimS* F | AGTAATGCTGCTCGTTTTGC |  |
| IE *fimS* R | GACAGAGCCGACAGAACAAC |  |
|  |  |  |
